# Fibrotic Scar After Spinal Cord Injury: Crosstalk With Other Cells, Cellular Origin, Function, and Mechanism

**DOI:** 10.3389/fncel.2021.720938

**Published:** 2021-08-26

**Authors:** Ziyu Li, Shuisheng Yu, Xuyang Hu, Yiteng Li, Xingyu You, Dasheng Tian, Li Cheng, Meige Zheng, Juehua Jing

**Affiliations:** Department of Orthopaedics, The Second Hospital of Anhui Medical University, Hefei, China

**Keywords:** spinal cord injury, fibrotic scar, crosstalk, cellular origin, treatment strategy

## Abstract

The failure of axonal regeneration after spinal cord injury (SCI) results in permanent loss of sensorimotor function. The persistent presence of scar tissue, mainly fibrotic scar and astrocytic scar, is a critical cause of axonal regeneration failure and is widely accepted as a treatment target for SCI. Astrocytic scar has been widely investigated, while fibrotic scar has received less attention. Here, we review recent advances in fibrotic scar formation and its crosstalk with other main cellular components in the injured core after SCI, as well as its cellular origin, function, and mechanism. This study is expected to provide an important basis and novel insights into fibrotic scar as a treatment target for SCI.

## Introduction

Fibrosis, which is defined as fibroblasts excessively depositing extracellular matrix (ECM) mainly composed of collagen (COL), fibronectin (FN), and laminin (LN), is a common reaction to injury and inflammation in the central nervous system (CNS) and peripheral organs (Bataller and Brenner, 2005; Lee and Kalluri, 2010; Travers et al., 2016; Lederer and Martinez, 2018; Mack, 2018; Dorrier et al., 2021). In the early phase of tissue injury, a fibrotic scar provides the necessary support structure for the injured area and maintains tissue integrity. Due to the persistent presence of fibrotic scar, the normal tissue structure is disordered, and organ function is affected to varying degrees (Rockey et al., 2015; Pardali et al., 2017; Dorrier et al., 2021). Therefore, interventions targeting fibrotic scar formation are expected to be a potential targeted treatment.

Spinal cord injury (SCI) results in a series of intricate pathological changes (Sofroniew, 2018; Tran et al., 2018). Generally, direct physical trauma leads to apoptosis and necrosis of various cells, which, in turn, triggers inflammatory and immune responses. Finally, the persistence of scar tissue and inflammatory cells hinders axonal regeneration and impairs functional recovery (Hara et al., 2017; Dias et al., 2018; Kobayakawa et al., 2019). The scar tissue that forms after SCI mainly includes astrocytic scar, fibrotic scar, and microglial scar (Bellver-Landete et al., 2019). After SCI, activated astrocytes gradually aggregate, overlap, and surround the edge of the injured core, forming an astrocytic scar that contributes to limiting inflammation and promoting tissue retention, ultimately inhibiting axonal regeneration and resulting in permanent functional deficits (Wanner et al., 2013; Lang et al., 2015; Anderson et al., 2016; Hara et al., 2017; Tran et al., 2018). Contemporaneously, fibroblasts gradually proliferate, migrate, and corral a large number of macrophages in the injured core, forming a fibrotic scar adjacent to the medial side of the astrocytic scar (Göritz et al., 2011; Soderblom et al., 2013; Zhu et al., 2015a). The fibrotic scar is thought to maintain tissue integrity, limit inflammation, and inhibit axonal regeneration after SCI (Klapka et al., 2005; Dias et al., 2018; Wang et al., 2018a,b). The microglial scar located between the astrocytic scar and fibrotic scar is another glial scar component that was recently proposed and contributes to limiting inflammation, promoting wound healing, and enhancing functional recovery (Bellver-Landete et al., 2019). In the injured core after SCI, the area occupied by fibroblasts is larger than that occupied by the microglia, and the number of fibroblasts is approximately twice that of astrocytes (Göritz et al., 2011; Bellver-Landete et al., 2019). The ablation of astrocytic scar or microglial scar exerts adverse rather than beneficial effects on inflammation resolution and wound contraction, while moderately attenuating fibrotic scar may dramatically facilitate axonal regeneration and functional recovery after SCI (Herrmann et al., 2008; Renault-Mihara et al., 2017; Wang et al., 2018a,b; Bellver-Landete et al., 2019). However, most studies on scar tissue have focused on fibrotic scar in neurodegenerative disease or on astrocytic scar in SCI, and fibrotic scar after SCI has received little attention (Zhang et al., 2005; Kostyk et al., 2008; D'Ambrosi and Apolloni, 2020). Recently, Corey R. Fehlberg et al. and David Oliveira Dias et al. reviewed the formation, origin, and function of fibrotic scar in the CNS, providing new perspectives for the treatment of SCI (Dias and Göritz, 2018; Fehlberg and Lee, 2021). However, the spatiotemporal distribution of astrocytes, fibroblasts, macrophages, and microglia is closely related after SCI, suggesting a complex crosstalk between the main cellular components and fibrotic scar in the injured core, which has not been reviewed.

Therefore, the present review aims to summarize novel and different perspectives for an understanding of the formation of fibrotic scar and its crosstalk with other main cellular components, as well as its cellular origin, function, and mechanism. This study is expected to provide an important basis and novel insights into the fibrotic scar as a treatment target for SCI.

## Fibrotic Scar Formation After Spinal Cord Injury

After SCI, fibrotic scar is mainly composed of fibroblasts and excess ECM, mainly including COL, FN, and LN (Camand et al., 2004; Okada et al., 2007; Schiwy et al., 2009; Soderblom et al., 2013; Zhu et al., 2015a; Yokota et al., 2017). However, the formation and distribution characteristics of fibrotic scar vary slightly among species or SCI models.

**Figure 1:**
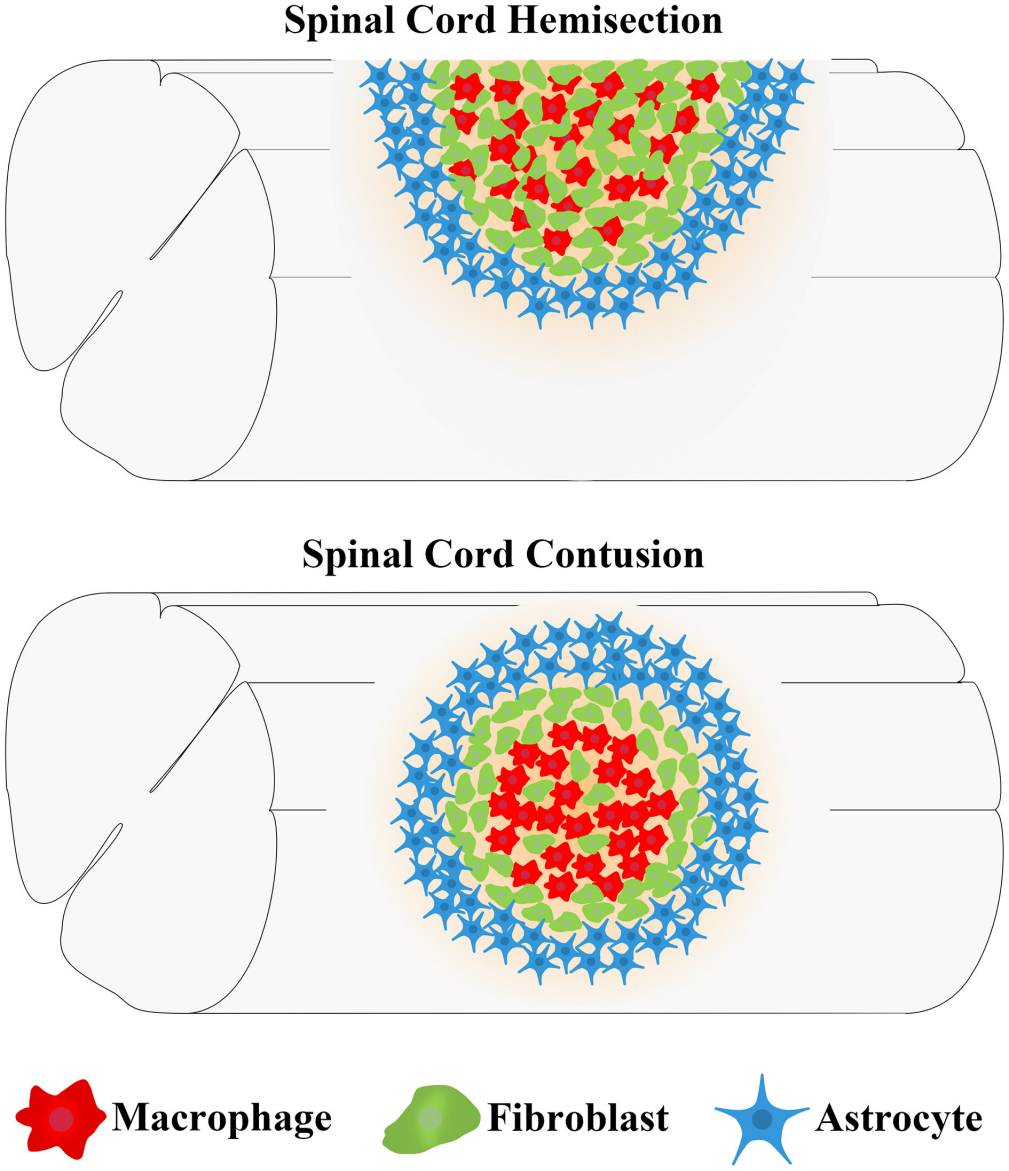
Differences in the locations of fibrotic scar between the spinal cord hemisection model and contusion model in mice. After spinal cord injury (SCI), fibroblasts form a fibrotic scar adjacent to the medial side of the astrocytic scar formed by astrocytes. The fibrotic scar after spinal cord hemisection injury fills the entire injured core and is mixed with macrophages, while the fibrotic scar formed after spinal cord contusion injury exhibits a dense and contiguous scar structure at the edge of the injured core that is filled with a large number of macrophages.

Due to high clinical similarity and high reproducibility, the mouse spinal cord hemisection model and contusion model are commonly used as the disease models of SCI (Soderblom et al., 2013; Dias et al., 2018; Bellver-Landete et al., 2019; Kobayakawa et al., 2019). In the spinal cord hemisection injury model, fibroblasts begin to proliferate, migrate, and aggregate in the injured area at 3 days postinjury (dpi) (Göritz et al., 2011). The number of fibroblasts increases more than 25-fold at 9 dpi and peaks at 14 dpi (Göritz et al., 2011). Meanwhile, fibroblasts aggregating in the injured area deposit a large amount of fibrous ECM and form a fibrotic scar adjacent to the medial side of the astrocytic scar, thus, filling the entire injured core, and the distributions of FN and LN overlap with fibroblasts (Göritz et al., 2011; Soderblom et al., 2013; Dias et al., 2018). As the fibrotic scar contracts, the number of fibroblasts begins to decrease at 4 months and then is maintained for at least 7 months after SCI (Göritz et al., 2011). In the spinal cord contusion model, similar to the spinal cord hemisection model at 3 dpi, fibroblasts begin to proliferate, migrate, and aggregate in the injured area (Göritz et al., 2011; Soderblom et al., 2013). The cell density increases significantly at 5 dpi and peaks at 7 dpi (Soderblom et al., 2013). In addition, as fibroblasts proliferate and aggregate, FN secreted by fibroblasts initially exists in a soluble form and begins to assemble into ECM at 7 dpi (Zhu et al., 2015b). At 14 dpi, fibroblasts deposit a large amount of fibrous ECM, such as FN, which becomes much more organized into a fibrillar network, forming a dense fibrotic scar adjacent to the medial side of astrocytic scar (Soderblom et al., 2013; Zhu et al., 2015b). However, unlike the fibrotic scar, which fills the entire injured core and is mixed with macrophages after hemisection injury, the fibrotic scar that forms after contusion injury exhibits a dense and contiguous scar structure at the edge of the injured core filled with a large number of macrophages (Figure 1; Göritz et al., 2011; Soderblom et al., 2013). In addition, the distribution of FN overlaps with fibroblasts, while the distribution of LN is mainly located around the peripheral rim of the injured core (Göritz et al., 2011; Soderblom et al., 2013; Zhu et al., 2015a; Dias et al., 2018; Li et al., 2021). In both injury models, fibroblasts begin to proliferate and migrate at 3 dpi and form mature and stable fibrotic scar structures at 14 dpi. Although differences in the spatial distribution of fibrotic scars in the two injury models have been identified, the similarity in the temporal distribution provides an important basis for subsequent research on fibrotic scar.

Consistent with the mouse spinal cord contusion model, the fibrotic scar also forms in a rat spinal cord contusion model, but the fibrotic scar distribution in the two species is slightly different (Ruschel et al., 2015; Zhu et al., 2015b). In contrast to mice, spinal cord contusion in rats leads to cavity formation in the injured core, which is considered to resemble the pathological changes observed in patients with SCI in the clinic (Metz et al., 2000; Norenberg et al., 2004; Buss et al., 2007). The existence of cavities after SCI in rats results in the fibrotic scars occupying a smaller area of the injured core than in mice, and the fibrotic scars are distributed along the edge of the cavity and partially overlap with astrocytic scars, indicating that astrocytes may be involved in the formation of fibrotic scars in rats and that differences may exist in the mechanism of scar formation between the two species (Zhu et al., 2015b). Considering the genetic homology with humans and the use of transgenic technology, a mouse model is still most commonly used to study SCI. However, advances in the treatment of SCI in mouse models should be further validated in rat models due to the cavity formation after SCI in rats, which is presumed to resemble patients with SCI in the clinic.

Overall, fibroblast proliferation and migration, and ECM deposition are important steps in fibrotic scar formation, and the temporal and spatial characteristics of fibrotic scar formation are of guiding significance for the formulation of subsequent research protocols. Studies aiming to further investigate the differences and commonalities of fibrotic scar among different species and injury models after SCI are very important to further understand the cellular origin, function, and mechanism of fibrotic scar, providing important bases for their use as a treatment target for SCI.

## Crosstalk Between The Fibrotic Scar And Other Cells After Spinal Cord Injury

**Figure 2:**
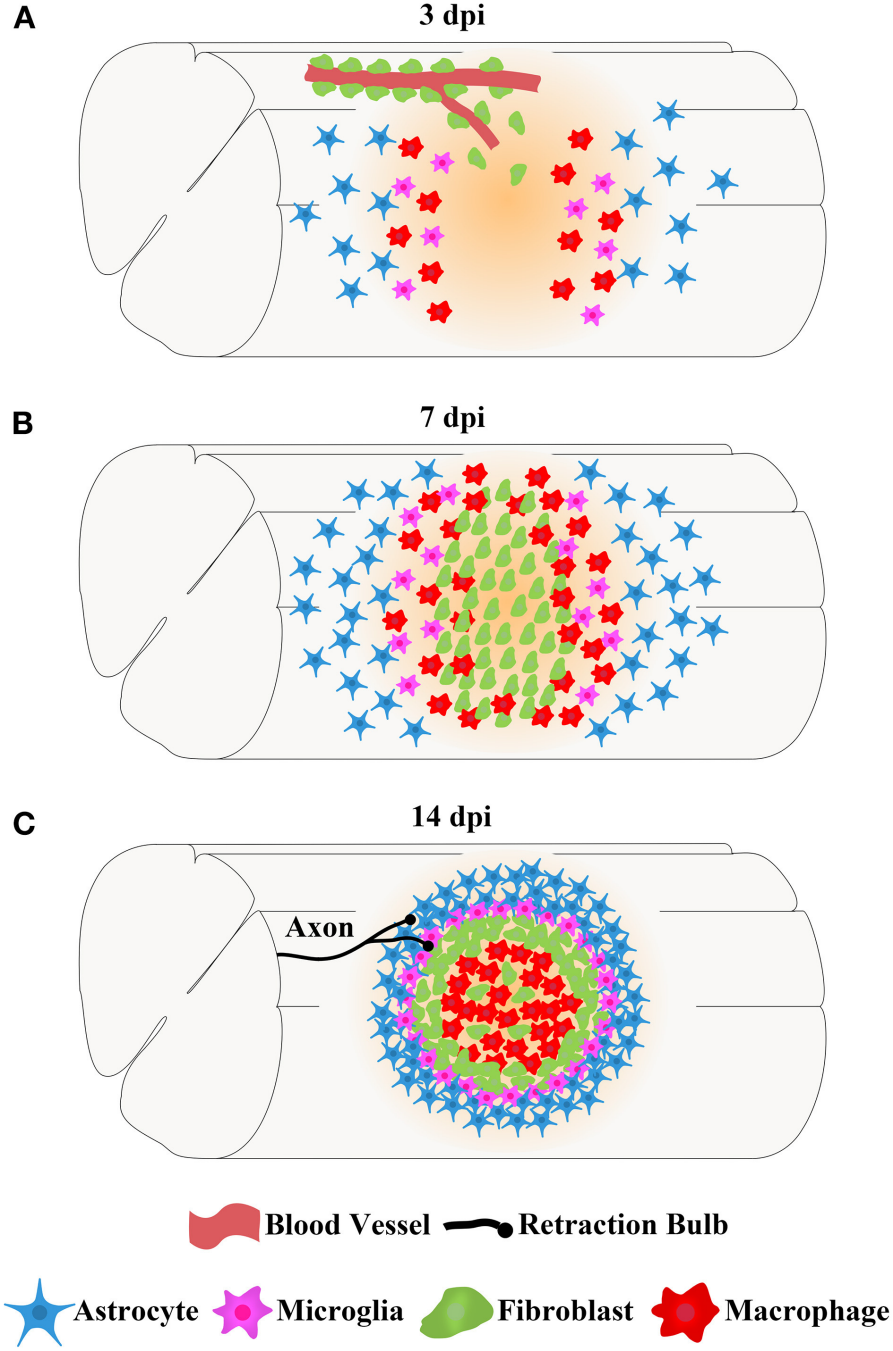
The spatiotemporal distribution of scars after spinal cord injury. After SCI, astrocytes, microglia, fibroblasts, and macrophages contemporaneously proliferate, migrate, and aggregate at the injured core. Finally, the cells work together to form complex, mature, and stable structures that fill the entire injured core, which is maintained in the chronic phase of SCI. **(A)** At 3 dpi, fibroblasts leave the blood vessels and are dispersed in the injured area together with astrocytes, microglia, and macrophages. **(B)** At 7 dpi, the numbers of fibroblasts, astrocytes, microglia, and macrophages are significantly increased, and these cells aggregate at the injured core. **(C)** At 14 dpi, astrocytes form the astrocytic scar that are distributed in the outer layer and surrounding the entire injured core. Fibroblasts form a fibrotic scar adjacent to the medial side of the astrocytic scar and corral the macrophages in the injured core. Microglia are located between the astrocytic scar and fibrotic scar, while macrophages are located at the most central site of the injured core. The axon tips form retraction bulbs after contacting fibroblasts and fail to penetrate the dense barrier formed by the fibrotic scar at the edge of the injured core, resulting in the failure of axonal regeneration.

The main cells in the injured area, including astrocytes, microglia, fibroblasts, and macrophages, work together to form complex, mature, and stable structures that fill the entire injured core and are maintained in the chronic phase of SCI (Soderblom et al., 2013; Zhu et al., 2015a; Anderson et al., 2016; Bellver-Landete et al., 2019; Zhou et al., 2020). The fibrotic scar is adjacent to the medial side of the astrocytic scar and corrals the macrophages in the injured core, while microglia are located between the astrocytic scar and fibrotic scar (Figure 2; Bellver-Landete et al., 2019). Studies focused on the closely related spatiotemporal distribution indicate a functional crosstalk between the main cellular components in the injured core (Dias et al., 2018; Bellver-Landete et al., 2019; Kobayakawa et al., 2019).

### Crosstalk Between the Fibrotic Scar and Astrocytes

Activated astrocytes deposit the ECM component chondroitin sulfate proteoglycans (CSPGs), forming an astrocytic scar that surrounds and limits the fibrotic scar and macrophages in the injured core after SCI (Wanner et al., 2013; Anderson et al., 2016). In particular, fibroblasts and astrocytes are adjacent to each other, suggesting that astrocytes may play an important role in the formation and function of a fibrotic scar. Signal transducer and activator of transcription 3 (STAT3) is a critical regulator of astrocytes, the formation of astrocytic scar, and corralling fibroblasts and macrophages after SCI (Herrmann et al., 2008; Wanner et al., 2013). GFAP–STAT3–CKO mice in which STAT3 is conditionally knocked out in astrocytes exhibit a loss of astrocyte hypertrophy and astrocytic scar disruption after SCI. Astrocytes at the edge of the injured core are dispersed and no longer overlap, resulting in the loss of the contiguous boundary of the fibrotic scar and the diffusion of macrophages (Herrmann et al., 2008; Wanner et al., 2013; Renault-Mihara et al., 2017). After the disruption of the astrocytic scar, the fibrotic scar alone does not completely limit the macrophages in the injured core after SCI, suggesting that studies based on limiting inflammation after SCI should consider the combined functions of the astrocytic scar and fibrotic scar. Moreover, Glast–Rasless transgenic mice in which the proliferation of fibroblasts is inhibited to attenuate fibrotic scarring after SCI exhibit a reduction in astrogliosis and disruption of the contiguous boundary of astrocytic scar (Dias et al., 2018). These results suggest a crosstalk between the astrocytic scar and fibrotic scar to maintain the structural stability of each scar type after SCI.

The ligand Ephrin-B2 and its cell membrane receptor EphB2 promote cell–cell contact and are bidirectionally activated via phosphorylation (Bundesen et al., 2003). Liza Q. Bundesen et al. reported that Ephrin-B2 expressed on astrocytes binds to EphB2 expressed on fibroblasts, triggering the formation of the boundary between astrocytic scar and fibrotic scar after SCI (Bundesen et al., 2003). Ephrin-B2^−/−^ mice in which the Ephrin B2 gene was deleted under the GFAP promoter exhibit a reduction in astrogliosis, facilitation of axonal regeneration, and improvement in motor function (Ren et al., 2013). Furthermore, after using RNAi to inhibit the expression of EphB2, the formation of astrocytic scar and fibrotic scar is inhibited, promoting axonal regeneration and myelination (Wu et al., 2021). These results indicate that the crosstalk between fibroblasts and astrocytes mediated by Ephrin-B2 and EphB2 is a therapeutic target after SCI, but specific intracellular signaling pathways require further exploration. In addition, Yona Goldshmit et al. reported that EphA4-deficient mice exhibit reduced astrocytic scarring, improved axonal regeneration, and better motor function after SCI (Goldshmit et al., 2004), in contrast to the report that mice lacking EphA4 do not exhibit a reduction in astrocytic scar or disruption of the boundary between astrocytic scar and fibrotic scar after SCI (Herrmann et al., 2010). The role of EphA4 in the crosstalk between astrocytes and fibroblasts requires further investigation.

### Crosstalk Between the Fibrotic Scar and Macrophages

Macrophages and fibroblasts are widely distributed in peripheral organs and the CNS, and crosstalk between macrophages and fibroblasts is involved in the maintenance of homeostasis under healthy conditions and in disease progression, especially the process of fibrosis (Cai et al., 2020; Buechler et al., 2021). However, the role and mechanism of macrophages in regulating fibrotic scar formation after SCI still remain to be explored. After SCI, blood-derived macrophages infiltrate the injured area and share many markers and behaviors with microglia, increasing the difficulty of distinguishing between macrophages and microglia (Wang et al., 2015). Recently, genetic fate mapping and conditional gene targeting technologies have allowed researchers to conclude that macrophages infiltrating from the circulatory system after SCI are mainly distributed in the injured core and limited by the fibrotic scar, while resident microglia are mainly localized at the lesion border and distributed between the astrocytic scar and fibrotic scar (Wang et al., 2015; Zhu et al., 2015a).

Macrophages and fibroblasts simultaneously proliferate, migrate, and aggregate at the injured core after SCI, and finally, a large number of macrophages fill in and are limited inside the mature fibrotic scar at 14 dpi (Zhu et al., 2015a,b). After the elimination of blood-derived macrophages in the injured core, the density of fibrotic scar is significantly reduced, and the contiguous boundary formed by the fibrotic scar is disrupted, suggesting that macrophages are involved in fibrotic scar formation by regulating fibroblast migration (Zhu et al., 2015a; Zhou et al., 2020). Further investigations of the cytokine expression profile after the elimination of blood-derived macrophages showed that the expression of a profibrotic molecule, a proliferation-inducing ligand (APRIL), was significantly decreased, while the expression of antifibrotic molecules and bone morphogenetic proteins (BMPs) was significantly increased (Zhu et al., 2015a). APRIL KO mice exhibit a reduction in the fibrotic scar area and an improvement in axonal regeneration after SCI, which may result from the reduced infiltration of B cells and macrophages (Funk et al., 2016). Therefore, the direct role of APRIL in the crosstalk between macrophages and fibroblasts requires further investigation, and BMPs and other potential molecular mechanisms should also be explored. In addition, integrin α5β1, which is mainly expressed on fibroblasts and macrophages, may be involved in the assembly of FN into the fibrotic scar by functioning as a receptor of FN after SCI, and the specific role of α5β1 requires further study to provide evidence for the crosstalk between macrophages and fibroblasts (Zhu et al., 2015b).

Similar to macrophages, neutrophils and T cells, which are inflammatory cells, are also involved in the inflammatory response after SCI, and their role in fibrotic scar formation should be considered in future studies (Li et al., 2018; Liu et al., 2019; Zivkovic et al., 2021). In a mouse multiple sclerosis (MS) model, with the infiltration of T cells, fibroblasts proliferate, aggregate, and deposit COL1 to form a fibrotic scar (Dorrier et al., 2021). The use of FTY720, which inhibits the exit of immune cells from lymph nodes and has been used to treat MS in the clinic, to inhibit the infiltration of immune cells, significantly results in the inhibition of fibroblast proliferation and COL1 deposition after MS (Dorrier et al., 2021). Therefore, the role of inflammatory cells other than macrophages in SCI, especially in fibrotic scarring, requires further attention. The effects of treatment strategies designed to suppress inflammation after SCI on the fibrotic scar should be considered, and further studies are needed to determine whether FTY720 and other CNS anti-inflammatory drugs that have been used in the clinic exert a therapeutic effect on SCI.

### Crosstalk Between the Fibrotic Scar and Microglia

During the period of fibrotic scar formation after SCI, microglia gradually distributed between the astrocytic scar and fibrotic scar, forming a microglial scar (Wang et al., 2015; Bellver-Landete et al., 2019). The special spatial distribution indicates that microglia may play a critical role in the formation of astrocytic scar and fibrotic scar. The use of PLX5622, a highly selective inhibitor of the colony-stimulating factor 1 receptor, to deplete the microglia disrupts the density and contiguous boundary of the astrocytic scar and fibrotic scar, resulting in the spread of macrophages in the injured core and the impairment of locomotor recovery after SCI (Bellver-Landete et al., 2019). Based on these results, the microglia are essential for maintaining the stability of scars. The migration of the microglia to the edge of the injured core to form scars is mediated by IGF-1, a microglia-derived factor, and the effect of IGF-dependent microglial migration on fibrotic scar formation after SCI and its mechanism require further exploration (Bellver-Landete et al., 2019).

Recently, Yi Li et al. reported that the microglia organize scar-free repair after SCI in neonatal mice, in significant contrast to persistent scars and failure of axonal regeneration in adult mice (Li et al., 2020). After SCI in neonatal mice, the microglia are the MG3 type, referred to as repair-promoting microglia that expressed proteinase inhibitors at high levels and transiently secreted FN to form effective bridges that connected the injured ends of the spinal cord. Finally, the nearly complete recovery of neonatal mice after SCI was observed, while macrophages, fibrotic scar, and astrocytic scar were absent (Li et al., 2020). After treatment with the chemical proteinase inhibitors E64 and serpinA3N and transplantation into the injured core of adult mice, adult mouse-isolated microglia exhibited a similar function to repair-promoting microglia, including less infiltration of macrophages, less deposition of COL, and more regenerated axons after SCI (Li et al., 2020). Therefore, repair-promoting microglia expressed higher levels of proteinase inhibitors, which have a critical role in resolving inflammation and inhibiting scar formation, thereby facilitating axonal regeneration. The role and mechanism of proteinase inhibitors in scar-free repair organized by microglia remain to be explored in both neonatal and adult mice, which may provide novel treatment strategies for SCI.

## Cellular Origin Of The Fibrotic Scar After Spinal Cord Injury

Previous electron microscopy studies have identified the presence of fibroblast-like cells in the contused spinal cord, and fibrotic scars have consistently been shown to form after SCI (Zhang et al., 2005; Kostyk et al., 2008). In the past, due to the limitation of a lack of specific markers for fibroblasts, the spatiotemporal distribution and origin of fibrotic scar-forming fibroblasts were unclear after SCI (Rudge and Silver, 1990; Shearer and Fawcett, 2001; Zeisberg and Kalluri, 2004; Darby and Hewitson, 2007; Krenning et al., 2010; Soderblom et al., 2013). Recently, using transgenic mice, Jonas Frisén et al. and Jae K. Lee et al. revealed the origin, phenotype, and distribution of fibrotic scar-forming fibroblasts after SCI (Göritz et al., 2011; Soderblom et al., 2013; Dias et al., 2018).

Pericytes, the perivascular cells associated with the microvascular system and wrapped around vascular endothelial cells may differentiate into fibroblasts and form a fibrotic scar in dermal scar and in kidney fibrosis (Sundberg et al., 1996; Lin et al., 2008; Humphreys et al., 2010). Jonas Frisén et al. used GLAST-CreER^T2^ transgenic mice to identify GLAST^+^ pericytes as type A pericytes and investigate their role in fibrotic scar formation after SCI (Srinivas et al., 2001; Slezak et al., 2007; Göritz et al., 2011). Blocking the proliferation of type A pericytes results in a significant reduction in, or even the disappearance of, fibrotic scar after SCI, suggesting that fibrotic scar-forming fibroblasts are derived from type A pericytes (Göritz et al., 2011). In the CNS, most or all pericytes are labeled with CD13, platelet-derived growth factor receptor (PDGFR) α and PDGFRβ, while some pericytes express desmin and α-SMA (Bondjers et al., 2006; Armulik et al., 2010; Daneman et al., 2010; Göritz et al., 2011). In the uninjured spinal cord, type A pericytes, which are enfolded by the plasma membrane of astrocytes and basal lamina, are distributed away from the blood vessel wall compared with type B pericytes and express CD13, PDGFRα, and PDGFRβ (Göritz et al., 2011). Type B pericytes express α-SMA or desmin (Göritz et al., 2011). After SCI, type A pericytes leave blood vessels, proliferate, and migrate to the injured area, depositing ECM to form a fibrotic scar. At this time, these cells no longer express CD13 and PDGFRα but express PDGFRβ and the fibroblast marker FN and transiently express α-SMA (Göritz et al., 2011). These results indicate that type A pericytes are the main source of fibrotic scar-forming fibroblasts that are specifically labeled with PDGFRβ after SCI.

Commonly used markers of fibroblasts, such as FN and COL, are components of the ECM and do not specifically label the cell body (Kalluri and Zeisberg, 2006). Jae K. Lee et al. used Col1α1-GFP transgenic mice to specifically label fibroblasts in the spinal cord and revealed that PDGFRβ^+^ perivascular fibroblasts are the main fibrotic scar-forming fibroblasts after SCI (Yata et al., 2003; Soderblom et al., 2013). In the uninjured spinal cord, Col1α1^+^ cells are mainly located around large-diameter blood vessels, and most of them surround smooth muscle cells in the arterioles, while 78.7% of Col1α1^+^ cells express CD13 and 95.7% of Col1α1^+^ cells express PDGFRβ but not desmin and α-SMA (Soderblom et al., 2013). After SCI, Col1α1^+^ cells leaving blood vessels proliferate, migrate, and aggregate at the edge of the injured core, depositing ECM to form a fibrotic scar around the injured core, where 26.9% of Col1α1^+^ cells express α-SMA, 26.9% of Col1α1^+^ cells express CD13, and 100% of Col1α1^+^ cells express PDGFRβ (Soderblom et al., 2013). As before injury, Col1α1^+^ cells still do not express desmin, α-SMA, or other markers of perivascular cells, including NG2, Olig2, and GFAP (Soderblom et al., 2013). Thus, Col1α1^+^ cells are perivascular fibroblasts forming a fibrotic scar that specifically express PDGFRβ after SCI (Zhu et al., 2015b; Funk et al., 2016).

Michael Vanlandewijck et al. performed a single-cell sequencing study that provided molecular definitions of blood vessels and blood vessel-related cells in the CNS of mice and revealed a population of PDGFRα^+^ fibroblast-like cells; many of the fibroblast-specific transcripts encode collagens, collagen-modifying enzymes, and proteins involved in collagen fibril assembly, indicating the fibrosis-promoting characteristic of fibroblasts (Vanlandewijck et al., 2018). PDGFRα^+^ fibroblasts are distributed around the large-diameter blood vessels in the meninges, parenchyma, and choroidal plexus (Vanlandewijck et al., 2018), consistent with the distribution of Glast^+^ or Col1α1^+^ fibroblasts reported by Jonas Frisén et al. and Jae K. Lee et al. (Göritz et al., 2011; Soderblom et al., 2013). However, PDGFRα^+^ fibroblasts are located between the vascular wall and astrocyte end-feet (Vanlandewijck et al., 2018), which is different from the study by Jonas Frisén et al., who reported that Glast^+^ fibroblasts are enfolded by the plasma membrane of astrocytes and the basal lamina (Göritz et al., 2011). Indeed, both Glast and PDGFRβ are expressed at high levels in fibroblasts and pericytes, suggesting that Glast^+^ fibroblasts and Col1α1^+^ fibroblasts are essentially perivascular fibroblasts, as reported by Vanlandewijck et al. (2018) and Dorrier et al. (2021). Recently, Cayce E. Dorrier et al. examined an MS model and reported that fibrotic scar is formed by Col1α1^+^ fibroblasts, which are initially distributed around large-diameter blood vessels and express PDGFRα and PDGFRβ (Dorrier et al., 2021). Although the localization of fibroblasts remains controversial, the fibrotic scar is formed from large-diameter perivascular fibroblasts that are labeled with CD13, PDGFRβ, and PDGFRα before injury and specifically labeled with PDGFRβ after SCI. Further study is needed to confirm this hypothesis.

## Function Of The Fibrotic Scar After Spinal Cord Injury

Scars, mainly astrocytic scar, fibrotic scar, and microglial scar, are important pathological changes in the chronic stage after SCI (Soderblom et al., 2013; Anderson et al., 2016; Bellver-Landete et al., 2019). The astrocytic scar has been widely studied and is considered to inhibit inflammation, maintain tissue integrity, and inhibit axonal regeneration (Anderson et al., 2016; Sofroniew, 2018; Xie et al., 2020). Indeed, the fibrotic scar also plays a dual function after SCI and should receive more attention (Hermanns et al., 2001; Göritz et al., 2011; Dias et al., 2018).

In the chronic phase of SCI, axon tips form retraction bulbs after contacting fibroblasts and fail to penetrate the dense barrier formed by the fibrotic scar at the edge of the injured core, resulting in the failure of axonal regeneration (Figure 2C; Dias et al., 2018). Nicole Klapka et al. reported that inhibiting TGFβ1 leads to delayed fibrotic scar formation after SCI, and regenerated CST axons pass through the damaged core, resulting in improved axonal regeneration and motor function (Klapka et al., 2005). Subsequent studies further confirmed that the reduction or disruption of fibrotic scar after SCI increases the density of regenerated axons in the damaged core, promoting the recovery of motor function (Hellal et al., 2011; Ruschel et al., 2015; Yokota et al., 2017; Cooper et al., 2018). Recently, Jonas Frisén et al. used Glast–Rasless transgenic mice to inhibit the proliferation of type A pericytes and specifically block pericyte-derived fibrotic scar after SCI; the results showed that attenuating fibrotic scar formation led to a reduction in astrogliosis, diminished astrocyte reactivity, disruption of the contiguous boundary of astrocytic scar, regeneration of 5-HT, and CST axons were enhanced, and the recovery of sensorimotor function was improved in the chronic phase of SCI (Dias et al., 2018). Therefore, the fibrotic scar significantly inhibits axonal regeneration in the chronic phase of SCI, and preventing fibrotic scar formation is an important direction for the treatment of SCI.

However, the inhibition of fibrotic scar formation did not significantly improve motor function despite the promotion of axonal regeneration in other studies (Zhu et al., 2015a). Jonas Frisén et al. showed that completely blocking the fibrotic scar results in the failure of wound closure and the spread of macrophages in the injured core at 14 dpi (Göritz et al., 2011; Dias et al., 2018). Therefore, in addition to inhibiting axonal regeneration in the chronic phase of SCI, fibrotic scarring also exerts beneficial effects on maintaining tissue integrity and limiting inflammation in the early phase of SCI. Treatment targeting the fibrotic scar should be based on the dual function of fibrotic scar in different phases of SCI, and interventions promoting the formation of a fibrotic scar in the early phase while inhibiting the formation of the fibrotic scar in the chronic phase may exert a better therapeutic effect.

## Mechanism Of Fibrotic Scar Formation After Spinal Cord Injury And Treatment Strategies

The fibrotic scar is recognized as a treatment target in many diseases, including SCI (Pardali et al., 2017; Dias et al., 2018; Dorrier et al., 2021). Therefore, a review of the mechanisms of the fibrotic scar formation may provide novel insights into the role of the fibrotic scar as a treatment target after SCI. Migration, proliferation, and ECM deposition are the key processes fibroblasts use to form fibrotic scar (Göritz et al., 2011; Soderblom et al., 2013; Dias et al., 2018), of which the mechanisms should receive more attention and be well-reviewed.

### Transforming Growth Factor Beta 1

Transforming growth factor beta 1 (TGF-β1) is a profibrotic factor that regulates fibroblast proliferation and ECM deposition in the CNS (Kawano et al., 2012; Wang et al., 2018a). The expression levels of microRNA-21-5p and TGF-β1 are significantly increased after SCI (Wang et al., 2018a). TGF-β1 promotes the expression of microRNA-21-5p and fibrosis-associated genes such as FN and COL in fibroblasts, while the upregulation of microRNA-21-5p enhances the profibrotic activity of TGF-β1 toward fibroblasts *in vitro* (Wang et al., 2018a,b). Based on these results, microRNA-21-5p enhances TGF-β1 activity in an amplifying circuit, promoting fibroblast-mediated formation of fibrotic scar after SCI. In addition, intrathecal injection of antagomir-21 was used to establish a microRNA-21-5p knockdown mouse model, resulting in improved functional recovery after SCI (Wang et al., 2018a,b). However, animal studies are needed to further confirm that microRNA-21-5p targets TGF-β1 to regulate the formation of a fibrotic scar after SCI.

Bradke et al. reported that SCI mice treated with the microtubule-stabilizing agents Taxol and epothilone B (epoB) exhibit enhanced axonal regeneration and the inhibition of fibrotic scar formation partially through dampening of the TGFβ1/Smad2 pathway, which is conducive to functional recovery after SCI (Hellal et al., 2011; Ruschel et al., 2015). Nicole Klapka et al. treated SCI rats with 8-Br-cAMP to inhibit the fibrotic effect of TGF-β1 and the iron chelator BPY-DCA to inhibit prolyl 4-hydroxylase, resulting in delayed COL IV expression and fibrotic scar formation (Hermanns et al., 2001; Klapka et al., 2005). The treatment improved axonal regeneration and motor function recovery (Hermanns et al., 2001; Klapka et al., 2005). In summary, TGF-β1 plays critical roles in fibrotic scar formation after SCI and can be used as a treatment target. Further studies examining the source of TGF-β1 and the molecular mechanism of the TGF-β1 pathway may be very important to reveal novel treatment targets for SCI.

### Periostin

Periostin (POSTN), an ECM protein, is a member of the fasciclin family that plays critical roles in the development of the heart, tooth, and bone tissue (Hakuno et al., 2010). POSTN also regulates fibrosis in many organs, such as the lungs, liver, and skin (Yamaguchi, 2014; Izuhara et al., 2016; Sugiyama et al., 2016). In particular, POSTN is specifically overexpressed in PDGFRβ^+^ cells referred to as fibroblasts after SCI, indicating that POSTN may regulate fibrotic scar formation after SCI (Yokota et al., 2017). In POSTN KO mice, the proliferation of fibroblasts is significantly inhibited after SCI, resulting in reduced fibrotic scarring, increased axonal regeneration, and improved functional recovery (Yokota et al., 2017). Further studies revealed that POSTN promotes macrophage infiltration to recruit fibroblasts to the injured area after SCI and increases the secretion of TNFα by macrophages to promote fibroblast proliferation (Yokota et al., 2017). Strikingly, mice receiving an injection of a POSTN-neutralizing antibody after SCI shows reduced fibrotic scarring and better functional recovery (Yokota et al., 2017). Thus, POSTN, specifically expressed in fibroblasts, is a potential profibrotic molecule that may promote fibroblast proliferation and migration, leading to fibrotic scar formation. Further studies investigating the role and molecular mechanism of POSTN in SCI may provide more evidence for targeted therapy for SCI.

### A Proliferation-Inducing Ligand

APRIL is well-known as a regulator of B-cell maturation and survival that is expressed in macrophages, B cells, and activated T cells (Chan et al., 2021). APRIL plays important roles in various diseases of peripheral organs and the CNS, such as rheumatoid arthritis and multiple sclerosis (Wang et al., 2012; Weldon et al., 2015; Funk et al., 2016). The depletion of macrophages in the injured core after SCI results in a reduction in the fibrotic scar density and the disruption of the contiguous boundary of fibrotic scar, promoting axonal regeneration (Zhu et al., 2015a). Therefore, treatment strategies that suppress inflammation may play additional roles in SCI by inhibiting fibrotic scar formation. In addition, after the depletion of macrophages, the reduction in fibrotic scarring is associated with the downregulation of APRIL, and the expression of APRIL and its receptor, B-cell maturation antigen (BCMA), is dramatically upregulated after SCI, suggesting that APRIL may be involved in fibrotic scar formation (Zhu et al., 2015a; Funk et al., 2016). In APRIL KO mice, the infiltration of B cells and macrophages is substantially reduced after SCI, resulting in a reduction in fibrotic scar area but no effect on fibroblast proliferation, and the effect of APRIL on macrophage and B-cell infiltration may be mediated by the upregulation of the expression of TNF-α and CCL2 (Funk et al., 2016). These results suggest that APRIL is a profibrotic molecule that is involved in the recruitment of fibroblasts by regulating the infiltration of macrophages and B cells after SCI.

### Fibronectin Including the Extra Domain A

The assembly of the FN matrix, a component of fibrotic scar, is an important step in the formation of a fibrotic scar after SCI, while the isoform of FN including the extra domain A domain (FnEDA) is involved in the pathogenesis of fibrotic scar in various diseases (Bhattacharyya et al., 2013; Zhu et al., 2015b; Cooper et al., 2018). The expression of FnEDA is significantly increased and that protein is chronically deposited in the injured core after SCI (Cooper et al., 2018). In FnEDA-null mice, the expression level of insoluble FN and the area of fibrotic scar are decreased significantly at 90 days after SCI, leading to improved axonal regeneration and functional recovery (Cooper et al., 2018). These results suggested a critical role for FnEDA in the assembly of fibrotic scar in the chronic phase after SCI and its potential use as a treatment target for SCI.

### Wnt

The Wnt pathway plays an indispensable role in disease processes in the CNS (Lie et al., 2005; Wang et al., 2011). The activity of Wnt signaling after SCI can be detected by performing X-gal staining in Wnt signaling reporter TOPgal transgenic mice (DasGupta and Fuchs, 1999; Yamagami et al., 2018). The Wnt/β-catenin pathway is transiently activated in FN^+^ fibroblasts in the injured core after SCI (Yamagami et al., 2018). Thus, the Wnt/β-catenin pathway may be involved in the formation of a fibrotic scar after SCI, which should be further investigated to provide a novel treatment target for SCI.

In addition, strategies such as neural stem cell (NSC) transplantation for SCI significantly promote axonal regeneration and functional recovery, suggesting that NSCs may be able to overcome the effects of adverse factors, including the fibrotic scar. The interaction between the two is worth further study. Mark H. Tuszynski et al. transplanted NSCs into rats or a primate model of SCI and showed that NSC-differentiated neurons extended axons through the injured core and filled the entire spinal cord segment, thus, forming new neuronal relay circuits and improving neural electrophysiological function and motor function (Lu et al., 2012; Rosenzweig et al., 2018; Kumamaru et al., 2019; Ceto et al., 2020). Although changes in the fibrotic scar have not been directly evaluated after NSC grafting, the regenerated axons passing through the injured core and filling the entire spinal cord segment suggest that NSCs may be able to overcome the inhibitory effect of the fibrotic scar (Lu et al., 2012; Rosenzweig et al., 2018). Nevertheless, Paul Lu et al. reported that human induced pluripotent stem cell (iPSC)-derived NSC grafts transplanted into the injured site differentiate into neurons that are distributed through most of the injured site, while a rift forms at the middle of the graft and is filled with collagen, thus, impairing axonal regeneration (Lu et al., 2014). This process results in the disconnection of neuronal relay circuits formed by the graft, and improved functional recovery is not achieved (Lu et al., 2014). These results indicate the need for additional studies of the changes in the fibrotic scar after NSC graft and its mechanism to provide the basis for the treatment of SCI by combining NSC grafts with inhibition of fibrotic scarring. Furthermore, Ephron S. Rosenzweig et al. reported that an injection of chondroitinase below the lesion to degrade CSPGs promotes axonal regeneration and improves motor function after SCI in primates, while the changes in the fibrotic scar are unknown (Rosenzweig et al., 2019). Studies targeting extracellular matrix molecules to treat SCI may focus on the effect on fibrotic scar, which is expected to provide a basis for the treatment of SCI.

**Figure 3:**
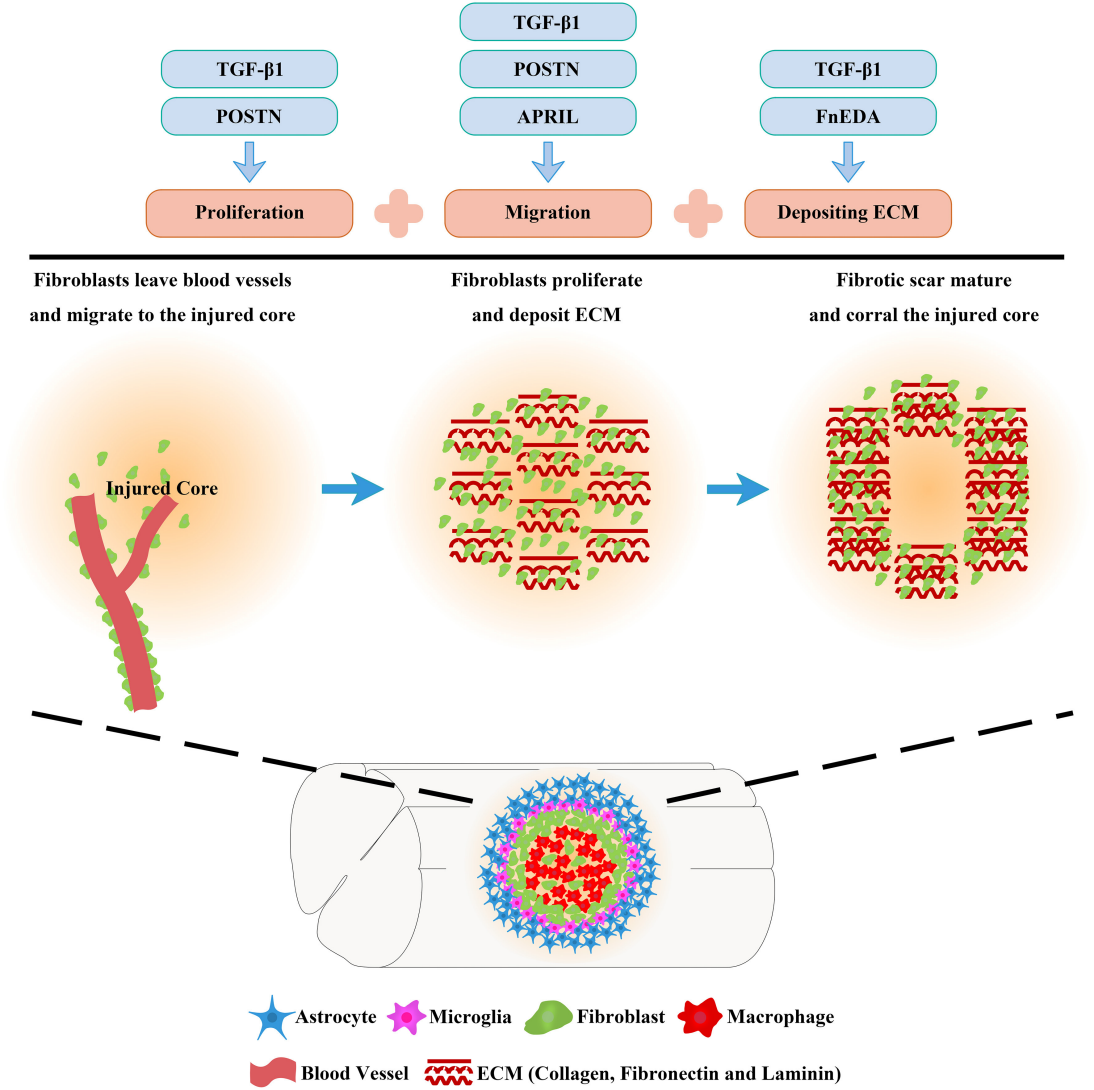
Molecular mechanisms of fibrotic scar formation after spinal cord injury. After SCI, fibroblasts leave blood vessels and then proliferate, migrate, and aggregate at the injured area, depositing extracellular matrix (ECM) to form fibrotic scar that corrals the injured core. Migration, proliferation, and ECM deposition are the key processes by which fibroblasts form the fibrotic scar, and the mechanisms can be used as treatment targets for SCI.

Overall, fibrotic scar formation after SCI is a complex process that includes the migration, proliferation, and ECM deposition of fibroblasts (Figure 3). The application of drugs and transgenic treatment strategies in an animal model of SCI has provided positive results that appropriately attenuating fibrotic scars contribute to axonal regeneration and the recovery of sensorimotor function (Table 1; Klapka et al., 2005; Zhu et al., 2015a; Yokota et al., 2017; Dias et al., 2018; Wang et al., 2018a,b). In addition, recent research advances in the identification and spatiotemporal distribution of fibrotic scar-forming fibroblasts after SCI have provided more evidence for the fibrotic scar as a treatment target after SCI. Further explorations of the cellular and molecular mechanisms underlying the formation of fibrotic scar after SCI are very important, which is expected to accelerate translation from the laboratory to the clinic.

**Table 1:** Treatments targeting the formation of fibrotic scar after spinal cord injury (SCI).

**Treatment strategy**	**Target**	**Effect on fibroblasts**	**References**
Antagomir-21	TGF-β1	Depositing ECM ↓	Wang et al., 2018a,b
EpoB	TGF-β1/Smad2	Migration and depositing ECM ↓	Ruschel et al., 2015
Taxol	TGF-β1/Smad2	Migration and depositing ECM ↓	Hellal et al., 2011
8-Br-cAMP	TGF-β1	Depositing ECM ↓	Klapka et al., 2005
POSTN-neutralizing antibody	POSTN	Proliferation and migration ↓	Yokota et al., 2017
APRIL KO mice	APRIL/BCMA	Migration ↓	Funk et al., 2016
FnEDA-null mice	FnEDA	Depositing ECM ↓	Cooper et al., 2018
Clodronate liposomes	Macrophage	Migration ↓	Zhu et al., 2015a
Glast-Rasless transgenic mice	Ras	Proliferation ↓	Dias et al., 2018

*EpoB, epothilone B; TGF-B1, transforming growth factor beta 1; ECM, extracellular matrix; POSTN, periostin; APRIL, a proliferation inducing ligand; BCMA, B cell maturation antigen; FnEDA, fibronectin including the extra domain A*.

## Author Contributions

JJ, MZ, and LC contributed to the design and revision of the review. ZL and SY edited and revised the manuscript. DT, XY, XH, and YL prepared the figures and table. All authors read and approved the final manuscript.

## Conflict of Interest

The authors declare that the research was conducted in the absence of any commercial or financial relationships that could be construed as a potential conflict of interest.

## Publisher's Note

All claims expressed in this article are solely those of the authors and do not necessarily represent those of their affiliated organizations, or those of the publisher, the editors and the reviewers. Any product that may be evaluated in this article, or claim that may be made by its manufacturer, is not guaranteed or endorsed by the publisher.
